# Laboratory-based determinants of simulated time trial performance in cyclists

**DOI:** 10.5114/biolsport.2023.122484

**Published:** 2023-04-06

**Authors:** Pedro L. Valenzuela, Lidia B. Alejo, Almudena Montalvo-Pérez, Carlos Revuelta, Diego Ojanguren, Alejandro Lucia, David Barranco-Gil

**Affiliations:** 1 Physical Activity and Health Research Group (PaHerg), Research Institute of Hospital 12 de Octubre (imas12), Madrid, Spain; 2 Department of Systems Biology, University of Alcalá, Madrid, Spain; 3 Faculty of Sport Sciences, Universidad Europea de Madrid, Madrid, Spain

**Keywords:** Prediction, Endurance, Cycling, Physiology, Testing

## Abstract

Different laboratory-based variables are individually associated with cycling performance, but scarce evidence exists on which of them, when all assessed in combination, could best explain cycling performance. The present study aimed to examine the combined association between laboratory-based endurance, strength/power and body composition indicators with time trial performance in high-level cyclists. Ninety-four male cyclists were recruited (age: 20 ± 3.5 years, maximum oxygen uptake [V̇O_2max_]: 77.7 ± 5.4 ml · kg^−1^ · min^−1^). Participants performed a maximal incremental cycling test for the assessment of endurance indicators (peak power output [PPO], V̇O_2max_, ventilatory threshold [VT] and respiratory compensation point [RCP]), and an incremental loading test to assess muscle strength and power-related outcomes (1-repetition maximum, mean maximal power) in the squat, lunge and hip-thrust exercises. Body composition was assessed by dual energy X-ray absorptiometry. On a separate visit, participants performed a simulated 8-minute time trial to assess cycling performance (determined as the mean power output attained). Strong-to-very-strong correlations were found between all endurance indicators and time trial performance (most r-values ranging between 0.68–0.92), whereas weaker correlations were found for strength/power (r-values < 0.5) or body composition (r-values < 0.7) indicators. Multivariate regression analyses revealed that VT, RCP and PPO explained together 92% of the variance in time trial performance (p < 0.001), with no significant contribution of the remaining variables. Although different endurance, strength/power and body composition individually correlate with simulated time trial performance in high-level cyclists, the former (and particularly VT, RCP and PPO) show the strongest association when all studied in combination. These findings underscore the importance of endurance capabilities (above strength/power or body composition) for maximizing time trial performance.

## INTRODUCTION

Endurance and, particularly, cycling performance is conditioned by a complex interplay between several factors, including race characteristics, tactics and environmental factors [1]. Chief among these factors is the individual’s fitness status, with three characteristics traditionally highlighted as the main determinants of endurance performance: the maximal ‘aerobic’ capacity of the individual (usually assessed through the determination of maximal oxygen consumption [V̇O_2max_] or peak power output [PPO] on an incremental test), the individual workload representing the boundary between heavy and severe intensity domains (which can be assessed through different markers such as critical power [CP], ventilatory threshold [VT], or the so-called lactate threshold), and exercise efficiency (i.e., the oxygen cost to generate a given power output [PO]) [2]. Indeed, several studies — although in most cases using small sample sizes and not analyzing professional cyclists as participants — support the use of laboratory-based endurance indicators (notably, V̇O_2max_, PPO, VT) as predictors of cycling performance [3–10].

Besides the aforementioned endurance indicators, growing evidence suggests that muscle strength/power also plays a major role in cycling performance. For instance, Kordi et al. recently reported that knee extension maximum voluntary torque was positively associated with both CP and with the amount of work completed above CP (known as W’, and considered as a marker of the so-called ‘anaerobic’ capacity) [11], and Cesanelli et al. reported a positive association between lower-limb maximal strength (i.e., 1-repetition maximum) and both the functional threshold power and the lactate threshold [12]. Indeed, strength training has proven beneficial for the improvement of not only muscle strength, but also of cycling and, particularly, time trial performance [13, 14], which seems to be partly due to beneficial effects on endurance indicators (e.g., PPO, VT) and neuromuscular variables (e.g., rate of force development, pedal stroke efficacy) [15–18]. However, other authors have failed to find a significant association between muscle strength measures (e.g., back squat 1-repetition maximum [1RM], knee extension maximum voluntary torque) and endurance indicators of cycling performance such as CP [19, 20].

Cycling performance can also be conditioned by other fitness-related factors, such as body composition. Increases in lean mass (and consequently body mass) have been traditionally regarded by cyclists as a detrimental adaptation because of the negative influence of gravity on performance (i.e., speed), particularly during uphill cycling [21]. In support of this, a recent study reported that performance improvements across one season in professional cyclists were mostly due to reductions in body mass, and not to increases in absolute PO values [22]. It must be noted, however, that a higher muscle mass can result in a higher absolute PO and speed, at least under those conditions in which gravity exerts a smaller influence (i.e., on flat roads). Indeed, quadriceps’ or thigh muscle mass has been identified as one of the main determinants of PPO and W’ in cyclists [19, 23–25]. Moreover, time trial performance has been reported to improve with strength training along with concomitant increases in muscle mass, with the improvement of both variables correlated [26]. Therefore, evidence overall suggests that body composition plays a major individual role on cycling performance. Yet, the interplay between body composition and endurance indicators in the prediction of cycling performance when assessed in combination remains unclear.

Although controversy exists in some reports, numerous variables seem therefore individually associated with cycling performance. However, which of these variables best explains cycling performance when all assessed in combination remains unknown, as physiological variables are usually interrelated and their interplay is complex [27]. For instance, it is possible that greater muscle strength levels or a larger muscle mass are associated with a higher cycling performance largely based on their indirect effects on endurance indicators (e.g., PPO, VT), but they might not actually predict cycling performance when assessed in combination with major endurance indicators. Identifying the physiological determinants of cycling performance is relevant, as it might help in performance prediction or talent identification, and could aid in guiding coaches and athletes in the design of training programs focused on those variables that appear more strongly correlated. Moreover, most studies to date have been conducted on recreational cyclists, with little evidence available on the predictors of performance in large cohorts of professional cyclists. The aim of the present study was to assess the individual and combined association between laboratory-based endurance, strength and body composition indicators and cycling performance (assessed through a simulated time trial), with an attempt to develop a multivariate model to predict elite cycling performance.

## MATERIALS AND METHODS

### Study design

Participants visited the laboratory on three days interspersed by 48 hours and approximately at the same time of the day (temperature = 20 ± 3°C, humidity = 25 ± 3%). They were instructed to maintain their normal dietary pattern and to refrain from intense exercise and consuming ergogenic aids/caffeine 48 hours before each testing session. In the first visit, participants underwent body composition assessment and a maximal incremental cycling test. During the second visit, they completed strength tests. A simulated 8-minute time trial was performed in the last visit (see below for further details).

### Participants

Ninety-four male road cyclists volunteered to participate in this study (aged 20 ± 3.5 years [range 16–37], body mass 63.9 ± 6.7 kg, V̇O_2max_ 77.7 ± 5.4 mL · kg^−1^ · min^−1^). All participants competed actively at the international or national level in the Professional, U23 or Junior categories. According to the guidelines proposed by de Pauw et al [28], these cyclists were overall considered to be of the highest performance level (i.e., Level 5). To be included in the study, cyclists had to be free of musculoskeletal injuries or other conditions that could hinder their participation. All participants were informed of the study procedures, benefits and risks and provided written informed consent. For those participants aged < 18 years, informed consent was signed by their parents or guardians. The study was approved by the Ethical Committee of the Alcorcón University Hospital (approval number 19/86). All procedures were conducted following the standards established by the Declaration of Helsinki and its later amendments.

### Procedures

#### Body composition

Height was measured using a wall stadiometer (Seca 437). Body composition (whole body fat and muscle mass, and bone mineral content) was measured by dual energy X-ray absorptiometry (DXA; Hologic QDR series Discovery; Bedford, MA). Assessments were performed at least two days after the last exercise session. Participants were recommended to maintain a similar eating and sleeping routine the day before each testing session and were advised to be euhydrated.

### Incremental cycling test

Cyclists performed a graded exercise test on their own bikes, which were placed on an indoor trainer (Hammer, CycleOps, Madison, WI) that has been proven valid to measure PO [29]. Participants started with a standardized 10-minute warm-up at 75 W, before completing a maximal incremental cycling test with an initial workload of 75 W, which increased by 5 W every 12 seconds (i.e., following a ramp-like protocol) until volitional exhaustion or when pedaling cadence fell below 60 rpm for more than 10 seconds. Gas exchange data were collected breath-by-breath (Ultima Series Medgraphics; Cardiorespiratory Diagnostics, Saint Paul, MN). The VT was determined as the workload at which an increase in both the ventilatory equivalent for oxygen (VE∙V̇O_2_^−1^) and end-tidal partial pressure of carbon dioxide (PetCO_2_) occurred with no concomitant increase in the ventilatory equivalent for carbon dioxide (VE∙VCO_2_^−1^). The respiratory compensation point (RCP, also termed ‘second ventilatory threshold’) corresponded to the work rate at which both VE∙V̇O_2_^−1^ and VE∙VCO_2_^−1^ increased together with a decrease in PetCO_2_ [30]. PPO was defined as the highest PO value reached during the test, and V̇O_2max_ was defined as the highest V̇O_2_ value (mean of 30 s) attained during the test.

### Muscle strength/power

Forty-eight hours after the incremental test, participants performed an incremental loading test to assess muscle strength and power-related outcomes in the squat, lunge and hip-thrust exercises. Exercises were performed on a Smith machine (Multipower Fitness Line; Peroga, Murcia, Spain). Bar mean propulsive power (MPP) during the concentric phase was measured with a validated linear position transducer (T-Force System; Ergotech, Murcia, Spain) [31]. The initial weight was 20 kg (i.e., only the bar), and the load was increased by 10 kg until a decrease in MPP was observed in two consecutive loads. Participants performed three consecutive repetitions with each load, and a 2-minute rest was allowed between loads. The highest MPP registered for each exercise was analyzed and 1RM was estimated as explained elsewhere [13, 14].

### Simulated time trial

During the third visit, cyclists performed an 8-minute time trial after a standardized 10-minute warm-up at 60% of their PPO. The simulated time trial was performed on the same bike and using the same aforementioned indoor trainer as for the incremental cycling test. Participants were instructed to attain the highest mean PO possible, and they were allowed to adjust resistance by changing the gears of the bicycle. They received no instructions regarding pacing and were blinded to PO values during the trial, and they were not allowed to stand on the pedals. An 8-minute time trial was chosen because all participants were familiar with this effort duration, it represents an effort commonly performed by cyclists during competition (e.g., during a Prologue), and it has been reported as a valid indicator of performance, being correlated with different laboratory-based performance indicators [32–34].

### Statistical analysis

Data are presented as mean ± SD. Normality (Kolmogorov–Smirnov test) and homoscedasticity (Levene’s test) of the data were checked prior to any statistical treatment. The relationship between endurance, strength and body composition indicators with simulated time trial performance (average W or W/kg) was analyzed with Pearson’s correlation coefficients (r); *r*-values of 0.1, 0.3, 0.5, 0.7, and 0.9 were considered small, moderate, strong, very strong and extremely strong, respectively [35]. Stepwise multivariate linear regression analysis was performed to determine the variables that best explained time trial performance. All variables were included in the model to avoid selection bias, and variance inflation factors (VIF) and tolerance values were checked to inspect for multicollinearity (i.e., VIF < 10 and tolerance > 0.1). Statistical analyses were performed with a specific statistical software package (SPSS 26.0, Inc., Chicago, IL) setting the alpha for significance at 0.05.

## RESULTS

Participants’ PO during the time trial averaged 347 ± 42 W (5.49 ± 0.5 W/kg). Strong-to-very-strong correlations were found between all endurance indicators and time trial performance expressed in both absolute (W) and relative units (W · kg^−1^) (*r*-values ranging from 0.68 to 0.93) (Table 1). By contrast, weaker correlation coefficients were found between time trial performance and strength/power (*r*-values < 0.5, Table 2) or body composition indicators (*r*-values < 0.7, Table 3).

**TABLE 1:** Association between laboratory-based endurance indicators and absolute (average W) and relative performance (average W/kg) in an 8-minute time trial

Predictor variable	Mean ± SD	Time trial (W)		Time trial (W/kg)	

		r	*p*-value	r	*p*-value
**PPO (W)**	430 ± 47	0.923**	< 0.001	0.343**	0.001
**Relative PPO (W/kg)**	6.80 ± 0.51	0.382**	< 0.001	0.863**	< 0.001
**V̇O_2max_ (L/min)**	5.02 ± 0.57	0.828**	< 0.001	0.256*	0.013
**Relative V̇O_2max_ (mL/kg/min)**	77.9 ± 5.4	0.458**	< 0.001	0.675**	< 0.001
**PO at RCP (W)**	367 ± 46	0.851**	< 0.001	0.381**	< 0.001
**Relative PO at RCP (W/kg)**	5.81 ± 0.58	0.340**	0.001	0.756**	< 0.001
**V̇O_2_ at RCP (%V̇O_2max_)**	92.4 ± 3.7	0.277**	0.007	0.284**	0.006
**PO at VT (W)**	261 ± 35	0.816**	< 0.001	0.495**	< 0.001
**Relative PO at VT (W/kg)**	4.14 ± 0.49	0.294**	0.004	0.762**	< 0.001
**V̇O_2_ at VT (%V̇O_2max_)**	70.6 ± 6.2	0.056	0.593	0.256*	0.013

Significant associations:

* *p* < 0.05;

** *p* < 0.01.

Abbreviations: PO, power output; PPO, peak power output; RCP, respiratory compensatory threshold; V̇O_2max_, peak oxygen uptake; VT, ventilatory threshold.

**TABLE 2:** Association between muscle strength/power indicators and absolute (average W) and relative performance (average W/kg) in an 8-minute time trial

Predictor variable	Mean ± SD	Time trial (W)		Time trial (W/kg)	

		r	*p*-value	r	*p*-value
**Squat 1RM (kg)**	83 ± 16	0.365**	< 0.001	0.104	0.320
**Squat relative 1RM [kg / body mass (kg)]**	1.30 ± 0.24	-0.004	0.972	0.238*	0.021
**Squat MMP (W)**	518 ± 121	0.436**	< 0.001	0.103	0.324
**Squat relative MMP [W / body mass (kg)]**	8.11 ± 1.67	0.136	0.191	0.228*	0.027
**Hip thrust 1RM (kg)**	101 ± 29	0.275**	0.007	0.000	0.997
**Hip thrust relative 1RM [kg / body mass (kg)]**	1.59 ± 0.45	0.203*	0.050	-0.067	0.523
**Hip thrust MMP (W)**	454 ± 121	0.264*	0.010	-0.027	0.796
**Hip thrust relative MMP [W / body mass (kg)]**	7.16 ± 1.93	0.180	0.083	-0.088	0.397
**Split squat 1RM (kg)**	58 ± 17	0.028	0.790	-0.240*	0.020
**Split squat relative 1RM [kg / body mass (kg)]**	0.91 ± 0.29	-0.047	0.655	-0.284**	0.006
**Split squat MMP (W)**	324 ± 107	0.002	0.988	-0.294**	0.004
**Split squat relative MMP [W / body mass (kg)]**	5.13 ± 1.82	-0.068	0.514	-0.327**	0.001

Significant associations:

* *p* < 0.05;

** *p* < 0.01.

Abbreviations: 1RM, one-repetition maximum; MMP, maximum mean power.

**TABLE 3:** Association between body composition indicators and absolute (average W) and relative performance (average W/kg) in an 8-minute time trial

Predictor variable	Mean ± SD	Time trial (W)		Time trial (W/kg)	

		r	*p*-value	r	*p*-value
**Body mass (kg)**	63.9 ± 6.7	0.696^**^	< 0.001	-.233^*^	0.024
**BMC (kg)**	2.26 ± 0.37	0.522**	< 0.001	-0.132	0.205
**BMC (%)**	3.56 ± 0.41	0.127	0.221	0.069	0.511
**Fat mass (kg)**	10.9 ± 2.1	0.262*	0.011	-0.451**	< 0.001
**Fat mass (%)**	17.1 ± 2.3	-0.114	0.275	-0.415**	< 0.001
**Muscle mass (kg)**	50.1 ± 5.1	0.740**	< 0.001	-0.136	0.190
**Muscle mass (%)**	79.2 ± 2.3	0.122	0.243	0.466**	< 0.001

Significant associations:

* *p* < 0.05;

** *p* < 0.01.

Abbreviations: BMC, bone mineral content.

**FIG. 1:**
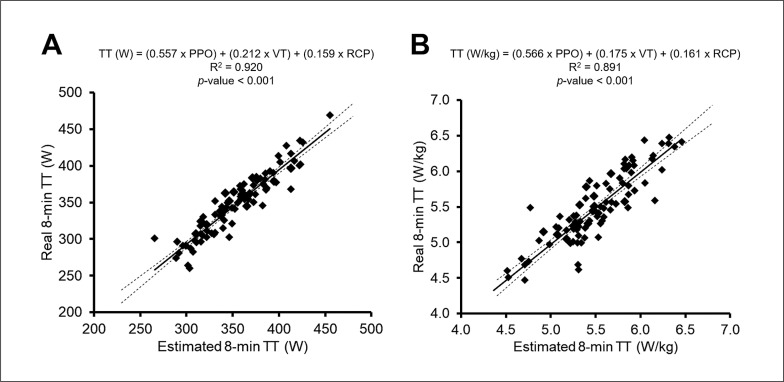
Association between the time trial (TT) performance estimated with the multivariate model and the actual time trial performance expressed both in absolute (average W, panel A) and relative (average W/kg, panel B) units. Note: Solid and dashed lines represent the line of identity and 95% confidence interval, respectively. Abbreviations: PPO, peak power output; RCP, respiratory compensation point; VT, ventilatory threshold.

Multivariate regression analyses including all variables as potential predictors showed that PPO, VT and RCP explained together 92% of the variance in time trial performance (all of them expressed in absolute units [W]) (R^2^ = 0.917, *p* < 0.001), with the three variables contributing significantly to the model (Figure 1, Table 4). No signs of strong multicollinearity were found for the variables included in the model (VIF and tolerance between 4.4–7.2 and 0.14–0.23, respectively). Similarly, the model including PPO, VT and RCP (in this case expressed in relative units [W/kg]) was also the one that best explained the variance in time trial performance when expressed in relative units (R^2^ = 0.887, *p* < 0.001), with the three variables contributing significantly to the model (Figure 1, Table 4). Again, no signs of strong multicollinearity were found for the variables included in the model (VIF and tolerance ranging between 4.2–5.9 and 0.17–0.24, respectively).

**TABLE 4:** Multivariate models predicting simulated time trial performance

Performance	Predictors	β (SE)	Standardized β	*p*-value	R^2^ (adjusted R^2^)	Model *p*-value
**Average W**	PPO (W)	0.557 (0.068)	0.649	< 0.001	0.920 (0.917)	< 0.001
	VT (W)	0.212 (0.077)	0.169	0.008		
	RCP (W)	0.159 (0.068)	0.172	0.022		

**Average W/kg**	PPO (W/kg)	0.566 (0.074)	0.649	< 0.001	0.891 (0.887)	< 0.001
	VT (W)	0.175 (0.082)	0.151	0.036		
	RCP (W)	0.161 (0.070)	0.176	0.024		

Abbreviations: β, regression coefficient; PPO, peak power output; RCP, respiratory compensation point; VT, ventilatory threshold.

## DISCUSSION

The main finding of the present study is that, while different laboratory-based endurance, strength and body composition indicators appear individually correlated with time trial performance, when all assessed in combination the former (and particularly PPO, VT and RCP) show the strongest correlation, explaining up to 92% of the variance in time trial performance in multivariate analysis. Strength and body composition indicators do not seem to further improve the predictive accuracy of the model.

A large body of knowledge supports the individual association between several laboratory-based endurance indicators and time trial performance. In the present study, we found that both V̇O_2max_ and particularly PPO – both indicative of cyclists’ maximal aerobic capacity – were strongly related to time trial performance, and the multivariate model indeed revealed that PPO was the variable that best predicted time trial performance. In line with this finding, previous evidence showed that PPO is strongly associated not only with V̇O_2max_ [8], but also with performance in time trials of different durations [4, 5, 8, 9]. For instance, strong correlations (*r* > 0.8) have been reported between PPO and 16-km [9], 20-km [8], 1-hour [4], or 90-minute time trial performance [7]. Of note, PPO has been reported to be more strongly correlated with time trial performance than V̇O_2max_ [4, 5, 7]. In addition to maximal aerobic capacity, the so-called ‘anaerobic’ threshold has been proposed to be a cornerstone of endurance performance [2], and our findings confirm this notion, with both VT and RCP showing strong correlations with time trial performance and contributing significantly to the multivariate model. This finding is supported by previous studies reporting strong correlations – stronger indeed that other indicators such as V̇O_2max_ – between threshold-related parameters (e.g., lactate or ventilatory thresholds) and time trial performance [4–7]. For instance, Nichols et al. [6] reported a stronger correlation between threshold-derived parameters and time trial performance than between V̇O_2max_ and time trial performance. In the same line, Lucia et al. found that PO at the VT, but not PPO or V̇O_2max_, was associated with performance in a 50-km time trial during the Tour de France [10]. Moreover, in line with our findings, Støren et al. found that PPO, V̇O_2max_. and the lactate threshold were strongly associated with performance on a 15-km time trial [36].

In contrast to endurance-related indicators, more controversy exists about the influence of strength/power on cycling and, particularly, time trial performance. There is evidence that maximal strength could be positively associated with cycling performance, at least in short-duration maximal efforts. For instance, lower-limb muscle strength has been positively associated with W’ [11, 19], which suggests that improving muscle strength could be beneficial for short-duration efforts (e.g., sprinting). In turn, there is mixed evidence on the association between maximum torque and CP, with the latter being more related to endurance performance. For example, Kordi et al. [19] reported that knee extensor maximum torque was associated with W’, but not with CP [19]. Byrd et al. [20] also found a significant association between back squat 1RM and W’, but no associations were found between back squat or other strength-related outcomes (e.g., knee extension torque at different angles) and CP. In support of these findings, we found overall weak correlations (*r* < 0.5) between strength/power indicators and time trial performance, and these variables did not significantly contribute to the multivariate model. In a group of regional and national-level road cyclists, Støren et al. found no association between strength/power variables (e.g., Wingate test, half squat test) and performance on a simulated 15-km time trial [36]. It must be noted, nonetheless, that while strength/power values might not be associated *per se* with cycling performance [36], resistance training has proven beneficial for cyclists, which seems to be partly due to a beneficial effect on neuromuscular variables (e.g., rate of force development, pedal stroke efficacy) [15, 16, 18]. However, the present findings suggest that the benefits of strength (and consequently of resistance training) should not overshadow the key role of endurance training for improving time trial performance.

Another major finding of the present study is that body composition parameters were overall weakly correlated with time trial performance, with the strongest correlations found between muscle mass (in kg) and absolute power output (*r* = 0.74). Supporting this finding, previous evidence suggests that muscle mass indicators (e.g., quadriceps or thigh muscle cross-sectional area or volume) are positively associated with power production capacity, at least for short-duration efforts (e.g., W’, PPO) [19, 23, 24]. In addition, strength training has been reported to improve cycling performance despite concomitant increases in muscle mass or muscle cross-sectional area [21], with changes in muscle cross-sectional area being positively correlated with improvements in time trial performance (assessed as relative PO) [26]. However, some debate exists on the association between changes in body composition and actual cycling performance. Although increases in muscle mass can covary with a greater PO production, an increased body mass might negatively affect riding speed due to the influence of gravity, particularly on the steepest roads. Indeed, relative PPO, and not absolute PPO, has been reported to better explain performance during uphill climbing [37]. Thus, whereas a decrease in fat mass is likely associated with an improved cycling performance, as confirmed in the present study by the negative association found between fat mass and relative power output, the association between muscle mass and performance remains unclear. Indeed, no associations were reported between muscle mass indicators and other markers of endurance performance such as CP [19]. Thus, our findings suggest that high absolute muscle mass values can be beneficial for absolute power production capacity regardless of fat mass, whereas the combination of low fat mass levels, along with high relative muscle mass levels (as a %), seems optimal for maximizing relative power output. Notwithstanding this, none of the analyzed body composition parameters contributed significantly to the multivariate model.

Some limitations of the present study should be acknowledged, notably its cross-sectional design, which precludes from drawing cause-effect conclusions. Indeed, despite the wide body of cross-sectional evidence suggesting a link between laboratory-based indicators and cycling performance, the scarce longitudinal evidence available suggests that these variables might have a limited accuracy for predicting future success in young cyclists [38]. Another potential limitation is that we assessed cycling performance through the PO attained on a simulated time trial in order to control, as much as possible, for all confounding variables (e.g., environmental conditions) but the assessment of speed on a real outdoor time trial could yield different results given the influence of other parameters such as aerodynamics (and consequently of body surface area). Indeed, the PO attained during a time trial is not necessarily associated with real performance (i.e., speed), and a previous study found that PPO was strongly associated with PO during a 16-km time trial, but not with the time needed to complete the trial [9]. Another limitation is the lack of control for potentially confounding variables such as motivation, or the absence of data on other important predictor variables such as exercise economy or muscle cross-sectional area, which have also been proposed as major indicators of cycling performance [2, 19, 23–25]. Moreover, the present findings might only be applicable to short-time trials, such as the one performed here (e.g., prologues, individual pursuit races), and might not necessarily be applicable to longer trials. It is also worth noting that the presence of multicollinearity cannot be completely ruled out, although the VIF and tolerance values observed (< 8 and > 0.14, respectively) suggest that there was not strong multicollinearity. Also, although we checked the linear association between numerous variables, future research should analyze whether nonlinear models can provide a better predictive accuracy. In turn, the large size (n = 94) and the high performance level of the analyzed sample can be considered major strengths, as well as the broad range of analyzed predictors.

## PRACTICAL APPLICATIONS

The present findings suggest that, although muscle strength and body composition indicators are individually associated with time trial performance, when multiple indicators are assessed in combination endurance ones are the strongest predictors, with other variables not meaningfully improving predictive accuracy. Particularly, given that PPO, VT and RCP explain most of the variance in time trial performance, our findings might support the routine assessment of these parameters for the monitoring of cyclists’ performance. Moreover, although the important role of muscle strength and body composition on cycling performance should not be disregarded, our results suggest that their benefits are mostly due to indirect effects on PPO, VT and RCP. Therefore, coaches and cyclists might preferably focus on the latter variables if their goal is to improve time trial performance, as these parameters appear as the main determinants.

## CONCLUSIONS

Different laboratory-based endurance, muscle strength and body composition indicators can be individually correlated with time trial performance, but the former present the strongest correlation and the combination of PPO, VT and RCP explains most of the variance (~92%) in time trial performance. These findings underscore the importance of endurance for predicting time trial performance, with muscle strength/power or body composition indicators *per se* not meaningfully improving the predictive accuracy.

## Conflict of Interest

The authors declare no conflicts of interest.
